# GSK461364A, a Polo-Like Kinase-1 Inhibitor Encapsulated in Polymeric Nanoparticles for the Treatment of Glioblastoma Multiforme (GBM)

**DOI:** 10.3390/bioengineering5040083

**Published:** 2018-10-09

**Authors:** Praveena Velpurisiva, Brandon P. Piel, Jack Lepine, Prakash Rai

**Affiliations:** 1 Department of Biomedical Engineering and Biotechnology, University of Massachusetts, Lowell, MA 01854, USA; Praveena_velpurisiva@uml.edu; 2 Department of Medical Oncology, Dana-Farber Cancer Institute, Boston, MA 02215, USA; Brandonp_piel@dfci.harvard.edu; 3 Biomolecular Characterization Lab, Core Research Facility, University of Massachusetts Lowell, Lowell, MA 01854, USA; Jack_Lepine@uml.edu; 4 Department of Chemical Engineering, University of Massachusetts, Lowell, MA 01854, USA

**Keywords:** GSK461364A, Glioblastoma Multiforme, polymeric nanoparticles, cytotoxicity, enhanced permeability and retention, polo-like kinase inhibitor, oncology, oncomedicine, U-87 MG

## Abstract

Glioblastoma Multiforme (GBM) is a common primary brain cancer with a poor prognosis and a median survival of less than 14 months. Current modes of treatment are associated with deleterious side effects that reduce the life span of the patients. Nanomedicine enables site-specific delivery of active pharmaceutical ingredients and facilitates entrapment inside the tumor. Polo-like kinase 1 (PLK-1) inhibitors have shown promising results in tumor cells. GSK461364A (GSK) is one such targeted inhibitor with reported toxicity issues in phase 1 clinical trials. We have demonstrated in our study that the action of GSK is time dependent across all concentrations. There is a distinct 15−20% decrease in cell viability via apoptosis in U87-MG cells dosed with GSK at low concentrations (within the nanomolar and lower micromolar range) compared to higher concentrations of the drug. Additionally, we have confirmed that PLGA-PEG nanoparticles (NPs) containing GSK have shown significant reduction in cell viability of tumor cells compared to their free equivalents. Thus, this polymeric nanoconstruct encapsulating GSK can be effective even at low concentrations and could improve the effectiveness of the drug while reducing side effects at the lower effective dose. This is the first study to report a PLK-1 inhibitor (GSK) encapsulated in a nanocarrier for cancer applications.

## 1. Introduction

Glioblastoma Multiforme (GBM) is an aggressive grade 4 malignant tumor arising from glial cells that affects the central nervous system [1]. Five-year survival rate is less than 5%, with a higher incidence in men than in women as mentioned in 2018 U.S. statistics [2]. This condition is majorly diagnosed in aged individuals with a smaller percentage among children [1]. The standard treatments in the clinic include a combination of chemo-radiation, that are associated with adverse side effects and makes a single form of chemotherapy ineffective in case of persistent GBM [3].

With the identification of new molecules to address the molecular targets often elevated in cancers, new classes of drugs are created to combat cancer. Polo-like kinases play a vital role in mitosis, especially during the transition from G2 phase to cytokinesis [4]. Deregulation of the serine/threonine kinase pathway and overexpression of PLK1 have been linked to tumorigenesis [5]. PLK1 inhibition has been shown to cause apoptotic cell death [6]. Among the list of selective PLK1 inhibitors, GSK, a targeted drug was shown to cause mitotic catastrophe by competing with the ATP binding site of PLK1 [3]. However, in the phase 1 clinical trials, it was concluded that GSK with its high specificity, inhibited PLK1 much better than other kinase inhibitors belonging to the same family. It exhibited significantly lesser neutropenia compared to its competitor, BI2536 [7]. However, in contrast to the toxicity profiles estimated and observed in preclinical studies, patients experienced higher venous thrombotic emboli (VTE) and sepsis at a dose of 225 mg, flagging it from further phases of clinical trials [7,8].

To circumvent these off-target effects and toxicity issues, this study was designed to encapsulate GSK in poly lactic-co-glycolic acid (PLGA), an approved biodegradable polymeric nanoparticle (NP) that enables controlled drug release [9]. Slow, low-dosage release of the drug product should, hypothetically, arrest cancer cell growth while minimizing toxicity to other tissues in vivo. Polyethylene glycol (PEG)-PLGA copolymer was used in the study since PEG extends the storage stability and retention time of the NPs in vivo [10]. This also extends the stability of the NPs in vitro. With the administration of these NPs encapsulating GSK, the particles will be retained in the tumor vasculature due to enhanced permeability and retention (EPR) effect [11]. The smaller size of these NPs (sub 200 nm) enable crossing of blood brain barrier (bbb) and delivery of the cargo [12].

**Figure 1:**
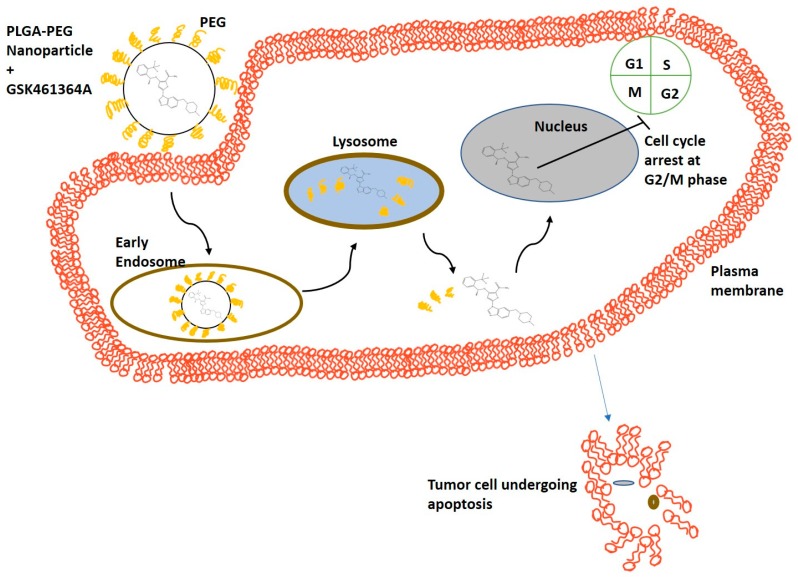
Schematic representing mechanism of action of cellular uptake of PLGA-PEG nanoparticles containing GSK461364A leading to apoptosis of tumor cells.

In this study, we tested these nanoconstructs in a primary tumor cell line, U87-MG, to observe time dependent cytotoxic effects of the free drug at various concentrations and compared these to that of the drug encapsulating NPs. Figure 1 demonstrates the potential mechanism of action of PLGA-PEG NPs encapsulating GSK, in the U87-MG cell line. Nanoparticle-based drug delivery to cancer cells is achieved through two mechanisms: (1) The drug is released from the nanoparticle that then passively diffuses across the cell membrane into the cytoplasm and (2) The cells via endocytosis take up the nanoparticles and the drug is released in the lysosomes. In either case, the free drug eventually causes cell cycle arrest in the G2/M phase.

## 2. Materials and Methods

Methoxy poly(ethylene glycol)-b-poly(lactide-co-glycolide) (mPEG-PLGA) (MW: 2,000–15,000 Da) and Poly(lactide-co-glycolide) (PLGA) (MW: 10,000–15,000 Da) were purchased from PolySciTech^®^ (West Lafayette, IN, USA). GSK461364A was obtained from APExBIO (Houston, TX, USA) and reconstituted in ethanol; BPD-MA (Verteporfin) was purchased from Sigma Aldrich (St. Louis, MO, USA); MTS assay kit was purchased from Promega Corporation^®^ (Madison, WI, USA), LIVE/DEAD^®^ Cell Imaging Kit was bought from Invitrogen™ (Carlsbad, CA, USA). Triton-X and other HPLC grade organic solvents were purchased from Fisher Scientific™ (Agawam, MA, USA). Annexin-V, Alexa Fluor™ 647 conjugate was purchased from Invitrogen™. Propidium iodide (PI), Annexin binding buffer and phosphate buffer saline (PBS) were purchased from abcam (Cambridge, MA, USA).

Human GBM cell line, U87-MG was purchased from ATCC^®^ (Manassas, VA, USA) and cultured in MEM alpha modification media with l-glutamine (Genclone™) with 10% fetal bovine serum (FBS) (Genclone™, San Diego, CA, USA), 1% PenStrep (Gibco™, Fisher Scientific) maintained at 37 °C, 5% CO_2_.

### 2.1. Synthesis and Characterization of GSK461364A Encapsulated in PLGA-PEG Nanoparticles

PLGA and PLGA-PEG nanoparticles with different ratios of PLGA and PEG (75:25 and 50:50), encapsulating GSK461364A and the empty nanoparticles (i.e., without the drug) were synthesized using nanoprecipitation method [13]. In summary, organic phase was prepared by adding 0.1195 mg of the drug dissolved in ethanol, 2.5 mg of PLGA or PLGA: PLGA-PEG (75:25) or in (50:50) ratio was dissolved in acetone, were added to a vial containing acetone. The drug concentration was measured using UV-Vis spectrophotometer based on the peak absorbance measured at 272 nm and 305 nm as shown in Supplementary Figure S1A. This organic phase was then added dropwise to a 2 mL of deionized water that acts as an aqueous phase. This mixture was left overnight at room temperature to let the organic solvents evaporate.

#### 2.1.1. Measurement of Size, Polydispersity Index (PDI) and Zeta Potential

The nanoparticles thus formed by self-assembly were subjected to ultracentrifugation using 30 KDa filtered centrifugal tubes (MilliporeSigma, Burlington, MA, USA) at 600 *g* for 15 min at 25 °C to remove non-encapsulated free drug and water. These nanoparticles were measured for their size, zeta potential and PDI using Malvern Zetasizer Nano ZS90 (Malvern Panalytical Inc., Westborough, MA, USA) based on the principle of Dynamic Light Scattering (DLS).

#### 2.1.2. Surface Morphology of the Nanoparticles

The morphology of the nanoparticles was better understood by performing Transmission Electron Microscopy (TEM). The nanoparticles were diluted with DI water and 3 µL of sample was added on carbon 200 mesh, copper (Electron microscopy sciences). The sample was air dried for 72 h and observed under TEM.

Similarly, 3 µL of diluted sample was added on a silicon wafer and air dried for 72 h, followed by sputter coating with gold to provide better conductivity of electrons and observed under scanning electron microscope (SEM).

#### 2.1.3. Measurement of Encapsulation Efficiency

The amount of drug encapsulated in PLGA-PEG nanoparticles was measured by adding 1% Triton-X and breaking open the nanoparticles. Thus, the drug concentration was measured using Nanodrop™ 2000c UV-Vis spectrophotometer (Thermo Fisher Scientific, Delaware City, DE, USA). The maximum wavelength (λmax) was measured at 311 nm as shown in Supplementary Figure S1B and the percentage of encapsulation efficiency was determined using the formula below.

% Encapsulation efficiency = ((Final amount of drug in mg)/(Initial amount of drug in mg)) × 100

### 2.2. In Vitro Stability

PLGA-PEG formulation containing the drug was stored in a vial covered in foil and stored at 4 °C for a week to study the stability of the nanoparticles for their shelf life. These nanoparticles were resuspended in 1:1 ratio in MEM alpha modification media containing 10% FBS and stored at 37 °C representing the stability under physiological conditions.

### 2.3. In Vitro Drug Release Kinetics

In vitro drug release studies were performed by dialysis bag diffusion technique [14]. This study was done in a beaker containing 800 mL of 1X PBS, pH 7.4 maintained at 37 °C and stirred at 150 rpm. A known volume of the sample with the known initial amount of drug present, was added to a regenerated cellulose dialysis bag of 20,000 Da MWCO (Spectra Max^®^, Chicago, IL, USA), with both the ends sealed. The beaker was covered with aluminum foil to prevent the heat loss and to prevent the exposure of the drug encapsulated nanoparticles to light. Samples were collected from the dialysis bag at different time intervals and equal volume of dissolution medium was added to the bag. These samples were used to measure the absorbance of GSK461364 at 311 nm using UV-spectrophotometer. This study was performed thrice on different days and the average values were used for plotting the release kinetics using DDSolver.

### 2.4. Cellular Uptake of Nanoparticles

U87-MG cells were seeded in a 35 mm culture dish at a density of 100,000 cells per dish and allowed for 6 h to adhere to the dish. Studies have shown the use of fluorescent hydrophobic model drugs such as Coumarin-6 to visualize cellular uptake and localization [15]. Since GSK461364A does not emit fluorescence, benzoporphyrin derivative monoacid (BPD), a hydrophobic drug of similar molecule weight, was used as a model drug to visually observe the cellular uptake of the nanoparticles under fluorescence microscope (EVOS™ FL Cell Imaging System, Life Technologies, Thermo Fisher Scientific). BPD encapsulated PLGA-PEG nanoparticles were synthesized and characterized in a similar fashion, as mentioned in earlier sections. The amount of BPD encapsulated was spectrophotometrically measured at 431 nm and 688 nm. The reasons behind using BPD as a model drug are due to its ability to fluoresce, similar molecular weight as GSK (718.79 and 543.6 respectively) and similar encapsulation efficiency as GSK when encapsulated in PLGA-PEG NPs. Thus, visualizing BPD containing PLGA-PEG NPs demonstrate the uptake of GSK in PLGA-PEG NPs. Cells were dosed with nanoparticles containing 500 nM of BPD and incubated for an hour at 37 °C, 5% CO_2_. As a control, equivalent concentration of free BPD dissolved in acetone was added. Other controls included, cells with no treatment and cells dosed with empty nanoparticles (without BPD). After an incubation of an hour, these dishes were washed with 1X PBS, pH 7.4 to remove any unbound drug and non-adherent cells. After the wash step, cells were added with media and observed under fluorescence microscope using Cy5 filter.

### 2.5. Determination of EC50

Effective Concentration (EC50) of GSK in U87-MG cells was determined by performing MTS assay, a colorimetric method to measure the cell viability. U87-MG cells were plated in a 96-well culture plate at a density of 10,000 cells per well and allowed to adhere to the surface overnight. Drug concentrations ranging from 1 pM to 17 µM were added and incubated for 72 h at 37 °C, 5% CO_2_. Controls for this experiment included live, untreated cells and dead cells (i.e., cells that were killed due to osmotic pressure by replacing growth medium with pure deionized water). After 72 h, to a final volume of 100 µL of media in the cells, 20 µL of PMS activated MTS reagent was added and incubated for 90 min. The absorbance was measured at 490 nm using a plate reader (Spectramax M2e (Molecular Devices, San Jose, CA, USA). The best fit model for the EC50 curve was obtained by using Dr Fit^©^ software (CRUK Cambridge Institute, University of Cambridge, Cambridge, UK) [16].

### 2.6. Live-Dead Assay

Cells were seeded at a density of 100,000 cells per well in a 35 mm culture dish and incubated overnight. The next day, cells were dosed with unencapsulated drug (free GSK) and drug encapsulated nanoparticles (nano GSK) with randomly chosen concentrations across the EC50 curve: 300 nM, 1 µM, 5 µM, 11 µM and incubated at 37 °C, 5% CO_2_ for 72 h. Live stain (calcein- AM) and dead stain (ethidium homodimer-1) (Live/Dead™ viability kit, Life Technologies Corporation, Thermo Fisher Scientific) were added at the specified ratios to a final volume of 1.5 mL and incubated at 37 °C, 5% CO_2_ for 90 min and the cells were observed under fluorescence microscope under transmitted, GFP and RFP filters.

### 2.7. Cytotoxicity Assay

To quantify cell viability after treatment, a standard MTS assay was performed. As described in the section on EC50 determination, the cells were dosed with the following concentrations of free and nano GSK (in an aqueous suspension): 300 nM, 1 µM, 5 µM, 11 µM, 15 µM and 17 µM and incubated at 37 °C, 5% CO_2_. The cytotoxicity assay was performed after 24 h, 48 h and 72 h in three different plates. The cell morphology was captured under the microscope at 24 and 48 h. On the other hand, viability was assessed for a short-term incubation for a concentration chosen in the higher range. A total of 100 µL of activated MTS reagent was added to each well and incubated for 90 min. Cell viability was determined at the end of the specified time points by measuring the absorbance using spectrophotometer at 490 nm.

### 2.8. Assessment of Cell Death Using Apoptosis Assay

To determine the mode of cell death caused by free as well as encapsulated GSK, apoptosis assay was performed. Cells with a density of 10^6^ per 35-mm culture dish were seeded and cultured in MEM-alpha modification media. Once the cells were adherent, they were dosed with 5 μM of free and nano GSK and incubated for different time periods. A time dependent study was performed to analyze the cell population in various stages of apoptosis or necrosis. After a set period of incubation, the samples were processed for flow cytometry using the procedure of Casciola-Rosen et al. and van Engeland et al. [17,18]. Samples were stained with Annexin-V/Alexa Fluor™ 647 conjugate (Invitrogen™, Carlsbad, CA, USA) and Propidium Iodide and observed under FlowSight^®^ imaging flow cytometer (Amnis^®^, EMD Millipore, Burlington, MA, USA) at emission spectrum of 642 nm and 488 nm respectively.

## 3. Results

### 3.1. Synthesis and Characterization of GSK461364A NPs

Empty and GSK NPs (with GSK encapsulated in various blends of PLGA and PEG containing nanoparticles) were characterized for their size, zeta potential and PDI using dynamic light scattering. Consistent hydrodynamic diameter values were obtained across different batches and an average of various repeats is represented in Table 1.

PLGA (100%) did not result in successful encapsulation of the drug, at times causing drug precipitation. To neutralize the excessive anionic charge on PLGA nanoparticle, we performed the synthesis using a mixture of PLGA and PEG. Based on the results described above, we continued to work with 75:25 PLGA-PEG blend, since it consistently yielded us with nanoparticles of desirable size and zeta potential, as excessive positive charge causes non-specific binding. More importantly, we obtained good encapsulation efficiency of the drug with this mixture. The size of the nanoparticles obtained in these experiments are sub 200 nm meaning that they can cross the bbb [12] and are suitable for further in vivo studies. A PDI value less than 0.15 indicates that there are no aggregates in the sample and that the sample has good size distribution. The negative zeta potential of −25.6 mV indicates that the nanoformulation is stable in a suspension.

**Figure 2:**
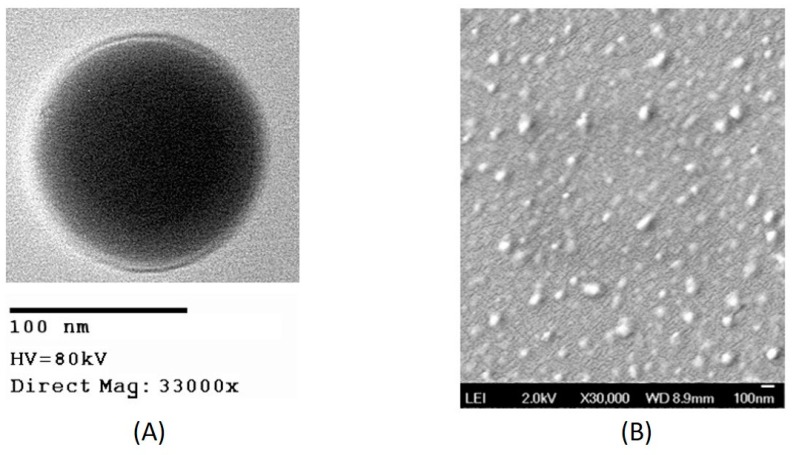
Images of GSK encapsulated in PLGA-PEG nanoparticles from (**A**) transmission electron microscope (**B**) scanning electron microscope.

The encapsulation efficiency of these particles was calculated by measuring the absorbance of the drug at 311 nm using UV-spectrophotometer, as shown in Figure S1B. An average of the encapsulation efficiencies obtained in different batches of synthesis is indicated in Table 1. As shown in the table, 56% of GSK that was initially added during synthesis was incorporated in the nanoparticles. The morphology of the drug encapsulated nanoparticles was confirmed to be spherical and the particles were distinct when observed under TEM as shown in Figure 2A and the size observed under TEM correspond to the hydrodynamic diameter measured using DLS, as expected. During the characterization of some nanoparticles, sometimes we observed an increase in size of NP when measured using DLS as shown in Table 1, which is due to the layer of the medium surrounding the nanoparticles when suspended in it [19]. The size of the nanoparticles observed under SEM, as shown in Figure 2B, was found to be consistent with the size observed under TEM as well as DLS.

### 3.2. In Vitro Stability of GSK461364A NPs

**Figure 3:**
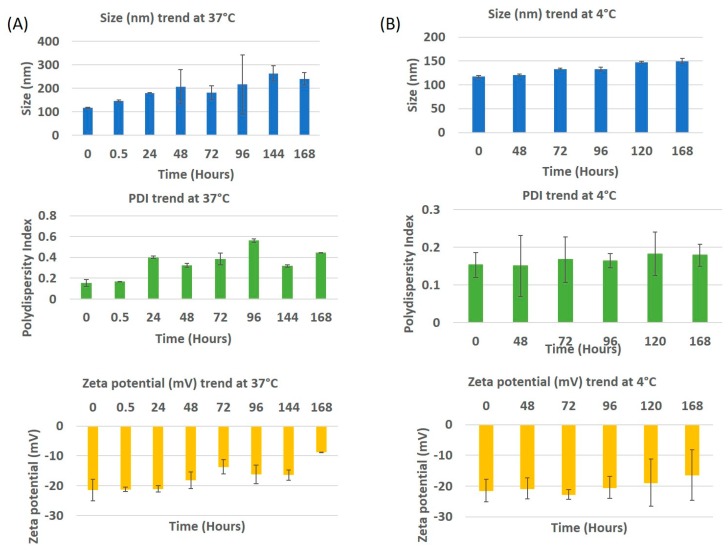
In vitro stability of drug encapsulated PLGA-PEG nanoparticles in media at (**A**) 37 °C and (**B**) 4 °C. The trends of size, PDI and zeta potential are indicated.

In vitro stability profiles of drug encapsulated PLGA-PEG nanoparticles maintained at 4 °C and 37 °C are indicated in Figure 3. In the case of the samples stored at 4 °C, an increase of ~30 nm in particle size was observed from day 0 to day 7, while the PDI remained almost the same until day 2 with an increase at day 3. This increase in PDI is explained by the aggregation of the nanoparticles after 72 h. The zeta potential did not change until day 4 with the value on day 0 being −21.4 mV, on day 5 it was −18.87 mV and day 7, −16.32 mV. This explains that the nanoparticles were stable at 4 °C. The stability can also be attributed due to the role of PEG [20].The stability study performed in media at 37 °C displays a drastic increase in the size and PDI after 30 min, that kept increasing until day 7 with a few minor variations. This steep increase in size and PDI may be due to proteins adsorbing to the surface of the nanoparticle, forming a corona [21]. To observe the impact on size, PDI and zeta potential, the study was performed until day 7. The size and PDI increased to ~250 nm and 0.45 respectively. The zeta potential slowly changed from being strongly anionic (−21.3 mV) to neutral (−8.88 mV), with time. This means that the colloidal nanoparticles became less stable with time when in media.

### 3.3. Release Profile of GSK461364A from PLGA-PEG Nanoparticles

**Figure 4:**
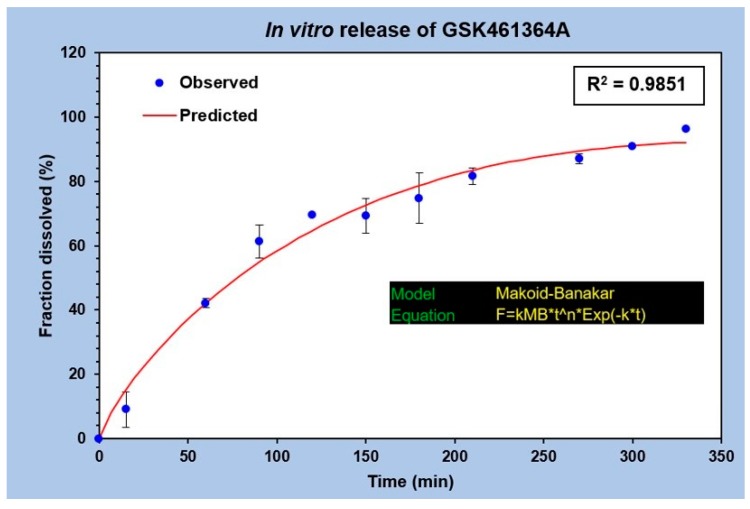
In vitro release profile of GSK461364A from PLGA-PEG nanoparticles performed in 1X PBS, pH 7.4 at 37 °C (n = 3).

The in vitro drug release profile of GSK was analyzed by plotting the fraction of drug released against time as shown in Figure 4. No burst release was observed in this study. A uniform and gradual drug release from t = 0 to t = 350 min was seen with the drug diffusing from the dialysis bag to the outside medium. A total of 50% of the drug release was observed between 60 and 90 min, while 80% of the drug release was observed at 210 min. This explains the slow drug release with the surface erosion of the polymeric nanocarrier under specified conditions. The graph in Figure 4 represents an average of three different release studies. The best fit curve of this regression model follows Makoid-Banakar model [22], with an R^2^ value greater than 0.98 and is represented by the equation,

F = kMB* t^n *Exp(−k*t),(1)

the kMB value of this release profile is 1.727, value of n is 0.81 and c being 0.002. Since the value of c is negligible, it can be assumed as zero, making the equation,

F = kMB* t^n,(2)

implying that it also follows the Korsmeyer Peppas model [23].

### 3.4. In Vitro Cellular Uptake of BPD Encapsulated Nanoparticles

Cellular uptake of PLGA-PEG nanoparticles containing BPD was observed under a fluorescence microscope. BPD encapsulated PLGA-PEG nanoparticles had a mean size of 93.58 nm with a PDI of 0.094 and a zeta potential of −37.9 mV.

**Figure 5:**
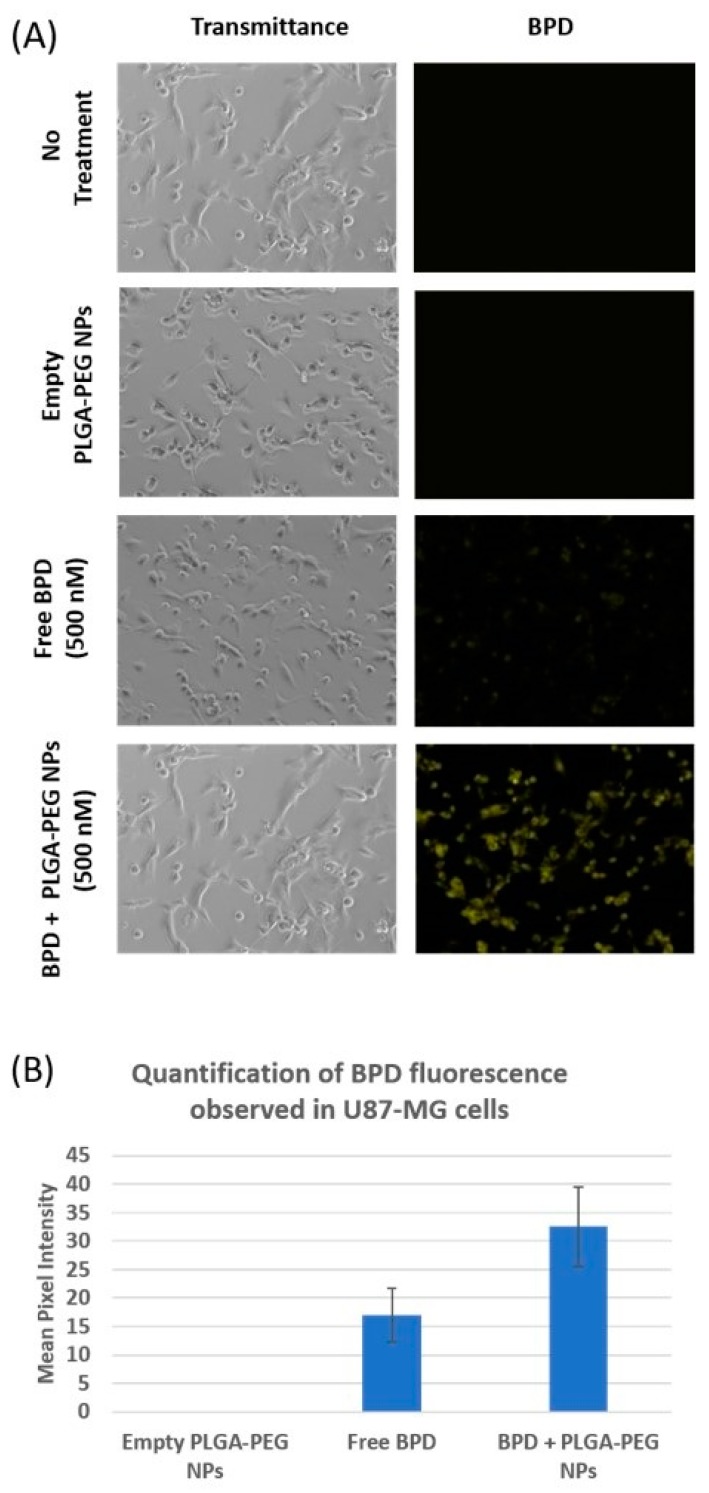
(**A**) Cellular uptake of 500 nM BPD encapsulated NPs captured under brightfield and Cy5 filters. Cells incubated with free BPD (500 nM) with two controls are also represented in this image: Empty PLGA-PEG NPs and cells with no treatment. (**B**) Mean pixel intensity of the fluorescence emitted by BPD in U87-MG is indicated comparing the empty PLGA-PEG NPs, free and nano BPD.

As shown in Figure 5A, no fluorescence was observed under cyanine 5 (Cy5) filter in cells which received no treatment as well as in those incubated with empty nanoparticles. Fluorescence was observed in the cells with free BPD treatment, but not as intense as those observed in cells dosed with the same concentration of BPD nanoparticles. The reason behind the brightest fluorescence in those cells incubated with BPD PLGA-PEG nanoparticles is most likely due to the enhanced permeability and retention (EPR) effect [11]. The fluorescence signal is also observed in the cells dosed with free BPD proportional to the intracellular concentration of the drug. The amount of drug observed inside the cells is the result of a net gain between the entry of drugs into the cells via fluid phase pinocytosis [24] and the efflux of drug across the cell membrane. These images, captured under fluorescence microscope, were analyzed using ImageJ software, and the histograms generated measure greyscale pixel intensity. As shown in Figure 5B, a substantial increase in mean pixel intensity was observed in the cells treated with nano BPD, corresponding to increased BPD fluorescence observed in the cells, compared to cells treated with free BPD. Cells that received no treatment as well as those treated with empty NPs showed no fluorescence.

### 3.5. EC50 Curve of Non-Encapsulated GSK461364 in U87-MG Cells

To determine the lethal dose of GSK461364A at which 50% of cell death is observed (EC50), cytotoxicity assay was performed. As shown in Figure S2, U87-MG cells were incubated with a wide range of concentrations of the drug ranging from 1 pM to 17 µM. Based on the average of eight repeats represented as these graphs, we identified two EC50 values for GSK in U87-MG cells, ~300 nM and ~(12–15) µM. Concentrations from 10 pM to 10 nM had a plateau curve at 100% viability indicating no effect of the drug on the cell line. This model provided an interpolated EC50 value of 0.198 µM. This model also computes the effect of free GSK on U87-MG cells resulting in 49.2% of viability at all concentrations from 300 nM until 17 µM, since the best fit curve flattens as seen in Figure S2. We further investigated the EC50 value in the nanomolar range (~300 nM) in its free and nano forms to compare the cytotoxicity effect that is similar to those observed with the second EC50 value.

### 3.6. Live-Dead Assay

Qualitative analysis of cell killing with free and drug encapsulated PLGA-PEG NPs was evaluated by live-dead assay.

**Figure 6:**
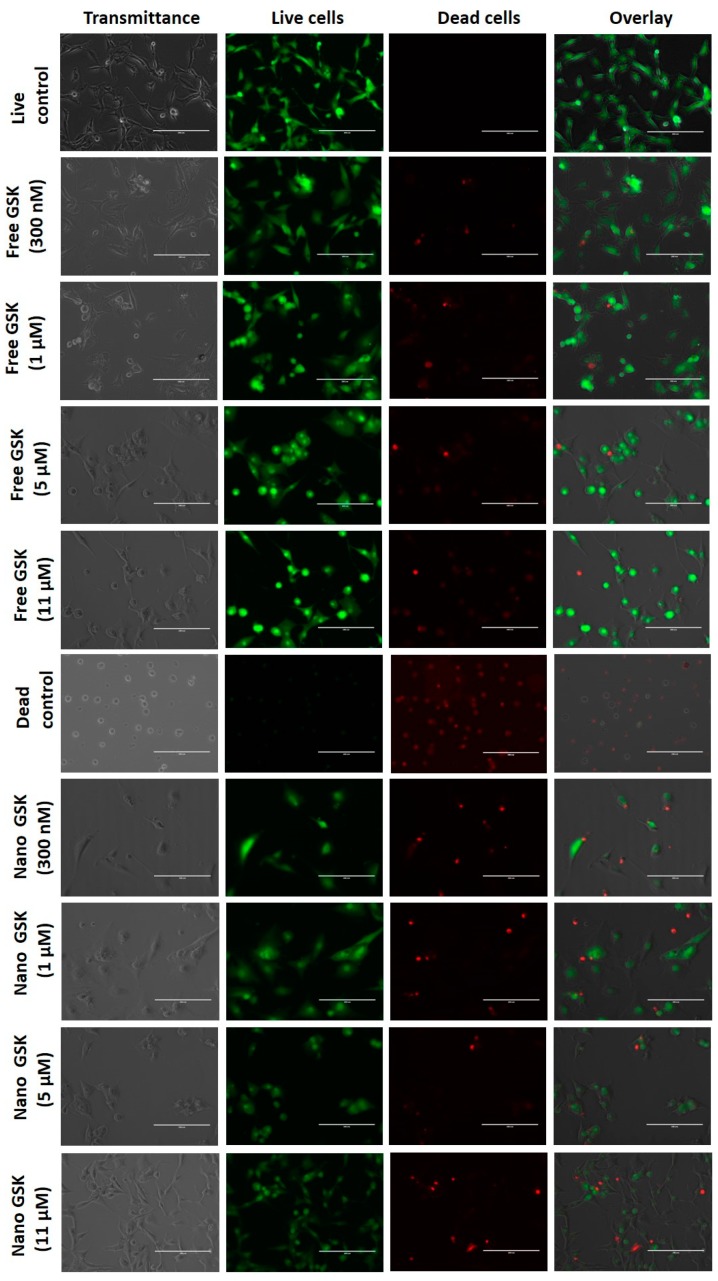
Live-Dead images of U87-MG cells treated with different concentrations of free and nano GSK. Images captured under transmittance, GFP (Live) and RFP filter (Dead) are indicated.

As shown in Figure 6, the live control and dead control are shown in the first and sixth rows respectively. This figure is divided into four columns, where the first one represents the brightfield image, the second column shows the number of viable cells, the third shows the number of dead cells and the fourth column is an overlay of live and dead cells. All these images were captured with the same intensity of light incident on the samples. Cells that received the treatment with free form of the drug at 300 nM and 1 µM show the maximum cytotoxicity among those in the free drug treatment arm. The images of cells treated with free 300 nM captured under transmittance and GFP filters show that there were relatively fewer viable cells compared to the live control. The morphology of the cells treated with 300 nM, 1 µM, 5 µM and 11 µM shifted from being spindle shaped to circular. Consistent with the viability values in the EC50 curve as well as the cytotoxicity assay, cells treated with 1 µM of free GSK had similar cell viability as those treated with free GSK of 300 nM, 5 µM and 11 µM.

The treatment arm with the drug encapsulated nanoparticles revealed higher cytotoxicity of the drug at low concentrations in the nanoparticle form compared to their free equivalents. Comparing 300 nM of GSK in the free and the nano form, we observed higher cell killing in the cells treated with the nanoparticle encapsulated GSK, with brighter red spots and fewer viable cells in green, compared to the free treatment. We also observed the density of viable cells to be visibly lower in the dish that received nano-encapsulated GSK treatment. Similarly, higher cell killing was observed in cells dosed with 1 µM of nano GSK compared to the free equivalents. This cytotoxicity trend aligns well with increased cellular uptake in the cells treated with the nano formulation of GSK as explained earlier in Figure 5. On the other hand, the morphology of the cells treated with 5 µM free GSK had a slightly higher number of circular cells as well as higher cytotoxicity than those incubated with the nano form. In the case of cells treated with 11 µM GSK, we found higher cell death compared to the free form. However, when cell morphology was observed, cells that received nano-encapsulated GSK had spindle shaped viable cells compared to the viable cells in the free drug arm, which were circular. In conclusion, higher cell killing was observed in the nano-encapsulated forms at 300 nM and 1 µM than their free forms; whereas at 5 µM and 11 µM, we observed higher cytotoxicity in the free drug compared to the nano treatment.

### 3.7. Measurement of Cell Viability, a Concentration and Time-Dependent Study

To confirm the cytotoxicity patterns observed qualitatively in the live-dead assay, a quantitative study was performed. U87-MG cells were dosed with various concentrations of GSK in the free and the nano forms and the cytotoxicity was measured using 3-(4,5-dimethylthiazol-2-yl)-5-(3-carboxymethoxyphenyl)-2-(4-sulfophenyl)-2H-tetrazolium (MTS) assay in a time dependent fashion.

**Figure 7:**
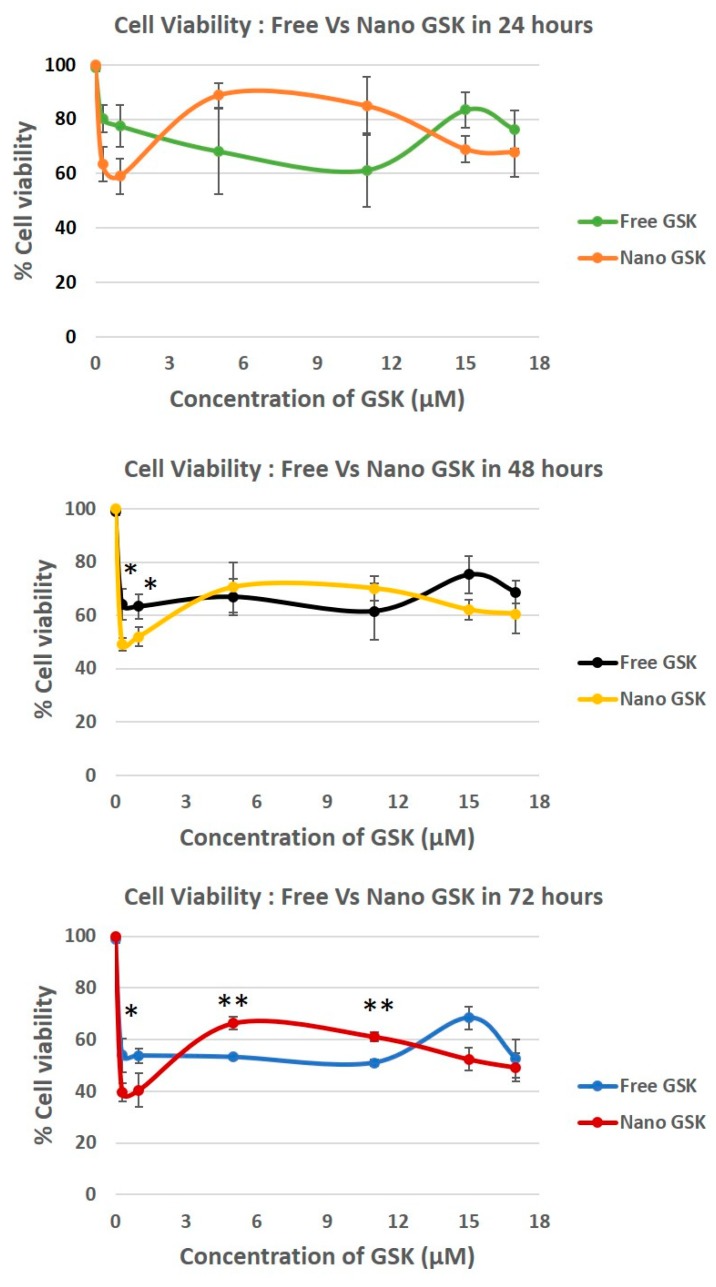
Time dependent cytotoxicity caused by different concentrations of GSK461364A in U87-MG cells post 24, 48 and 72 h. All viability values were normalized against the live control (no treatment). * *p* < 0.05, ** *p* < 0.005.

Both immediate and delayed cytotoxic effects of the drug were observed in U87-MG cells. As shown in Figure 7, delayed effects were observed at 24, 48 and 72 h. We observed a time dependent killing in all the concentrations tested. In the case of the cells treated with 300 nM and 1 µM, a statistically significant cytotoxicity of nano-encapsulated vs free drug treatment was observed at 48 and 72 h at 300 nM and 15 µM. This trend is suggestive of the nanoparticles being entrapped in the tumor cells possibly due to the EPR effect, thereby increasing exposure time and increasing the cytotoxicity of GSK.

Although there was no significant decrease in the cytotoxicity in 17 µM, nano GSK treated cells had reduced viability compared to the free form. Interestingly, a reverse in viability pattern was observed in those treated with 5 µM and 11 µM, reinforcing our data from the live-dead assay. A statistically significant cell killing was observed at 72 h in the cells treated with the free form of GSK at 5 and 11 µM compared to their nano counterparts. Cell viability for every concentration in both the free or nano-encapsulated form decreased with time, implying that the PLK1 inhibition is time dependent. It can also be suggested that the toxicity of the drug is not minimized due to the encapsulation in nanoparticles. Despite the drug release within 6 h, the effect of the drug takes at least 48 h to observe a significant change in the cell viability. Dosage with empty NPs did not have any effect on cell viability, with % viability being equal to no treatment.

**Figure 8:**
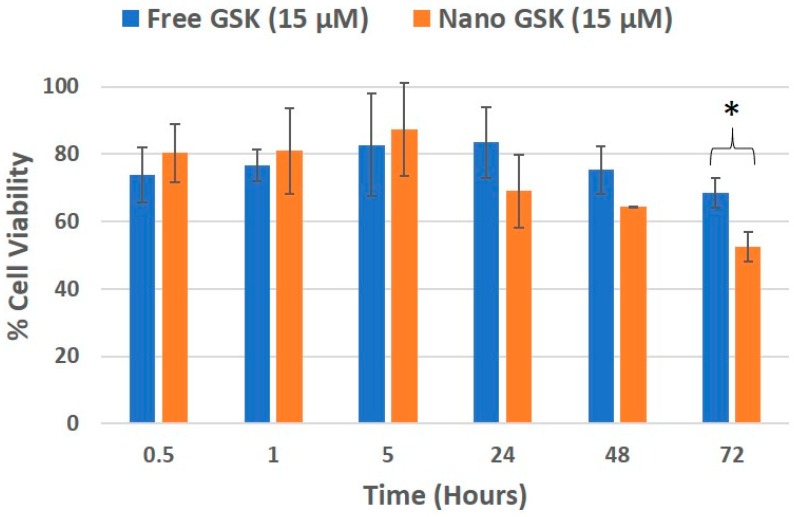
Cell viability trend of U87-MG treated with 15 µM GSK, that corresponds to the release profile. * (*p* < 0.05).

Cell viability was evaluated in a time and concentration dependent manner. At 300 nM GSK, the EC50 value was reached with the free drug at 72 h and nano GSK at 48 h. Similar results were seen at 1 µM GSK. At 15 µM and 17 µM, the EC50 was reached at 72 h for nano GSK. In conclusion, this study shows that GSK when encapsulated at a nanomolar concentration, can lead to 50% viability at 48 h. This highlights the need to eliminate administration of higher doses of GSK and also prevent off-target effects caused by the free form of the drug. To further understand the immediate effect of the drug, 15 µM GSK was randomly chosen of the concentrations tested earlier and the viability values were measured at 30 min, 1 and 5 h. This study was continued to understand the delayed drug effects seen at 24, 48 and 72 h. As shown in Figure 8, the viability values of U87-MG when treated with free and nano GSK remained similar until 5 h. This corresponds to the drug release pattern observed within 6 h as shown in Figure 2. At 24 and 48 h, there is a noticeable reduction in cell viability of those treated with nano GSK. Whereas at 72 h, a significant increase in cell killing was observed in cells treated with nano GSK compared to the free drug. This means that the free drug passively diffuses into the cell and inhibits PLK-1, but the effect fades out due to the free drug efflux, whereas the nanoparticles taken up by the cells via endocytosis will be retained.

**Figure 9:**
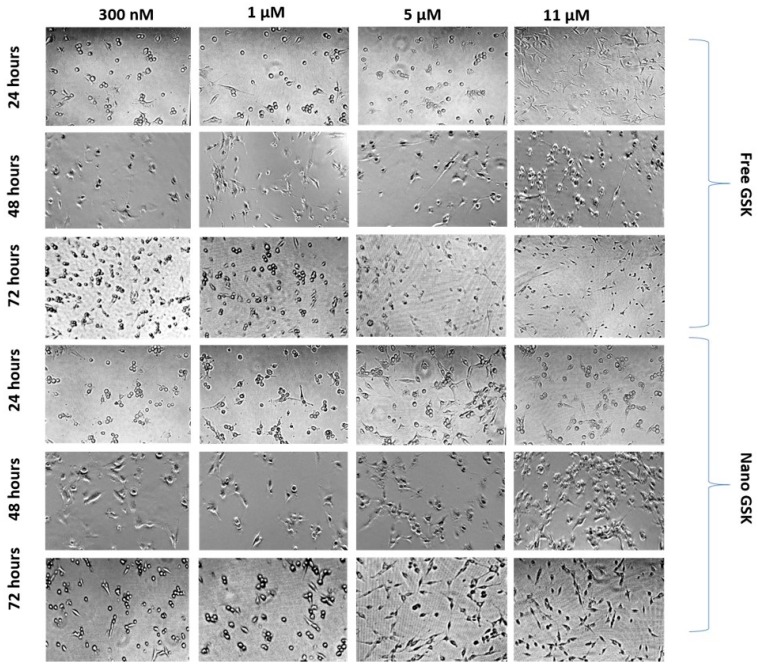
Morphological changes observed in U87-MG post treatment with free and nano GSK at 24, 48 and 72 h. The first three rows represent free GSK treatment and the bottom three indicate morphology of U87-MG cells post treatment with nano GSK.

While assessing the viability of the cells subjected to different treatment arms, morphology of the cells treated with a few concentrations was observed at 24, 48 and 72 h. As shown in Figure 9, cells subjected to 300 nM of nano GSK had many circular cells compared to the free treatment. A time dependent change in morphology was observed with the shape of the cells transitioning from fibroblast-like shaped to spherical. Cells with round morphology represent non-viable cells that are mostly non-adherent. Similarly, morphology of the cells that received 1 µM nano GSK had a higher number of circular cells compared to the free treatment. On the other hand, many rounded cells were observed in the cells treated with 5 µM and 11 µM free GSK at 72 h than nano GSK. The density of cells in the well that received free GSK (5 µM and 11 µM) was sparse, since there were more dead, non-adherent cells. Time dependent changes in morphology can be distinctly seen in those treated with 11 µM free GSK at 24 h, with the cells possessing typical U87-MG morphology. They assumed spherical shape at 48 h, with the highest dead cell populations forming post 72 h. From this experiment, we conclude that the morphological patterns remain consistent with the viability values quantitatively analyzed through MTS assay.

### 3.8. Evaluation of Cell Death via Apoptosis

**Figure 10:**
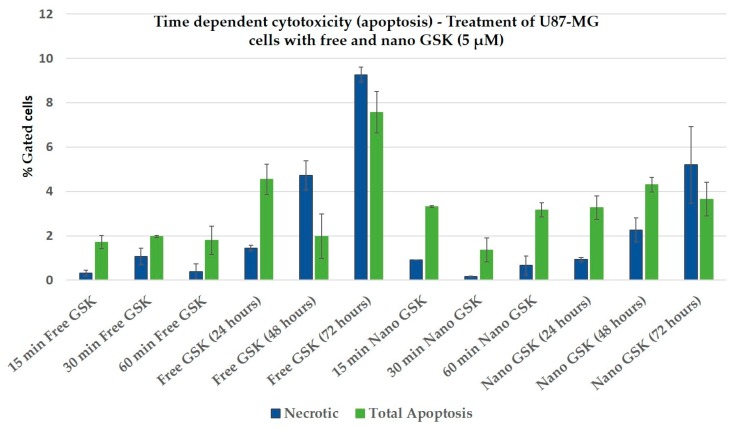
Bar graphs showing the percentage of apoptotic and necrotic cells following treatment with either free GSK or nano GSK at a drug concentration of 5µM. The percentage of necrotic as well as apoptotic cells from the “no treatment” control were subtracted from all of these treatment arms and plotted here. Error bars indicate standard deviation.

Apoptotic assay shows that the total apoptosis was higher than necrosis as shown in Figure 10, with short incubation times such as 15, 30 and 60 min. Except for free GSK at 48, 72 h and nano GSK at 72 h, we found the apoptotic death to be predominant. In these three cases, it can be said that secondary necrosis was observed, where the cells died initially via apoptosis, but after a certain point the plasma membrane was compromised. The graph below shows that there was more cytotoxicity (apoptosis and necrosis) caused by free GSK compared to nano GSK at 24 h, while at 48 h percentage of dead cells from free and nano GSK treatment coincide. Even at 72 h, percentage of total dead cells (apoptotic + necrotic) in the figure below states that at 72 h free GSK (5 µM) is more cytotoxic to U87-MG cells than nano GSK.

This trend at 24, 48 and 72 h exactly matches with the cytotoxicity quantitatively measured using MTS assay (Figure 7). Thus, the study confirms the killing patterns observed across live-dead assay (ethidium homodimer), cytotoxic assay (MTS) as well as the morphological examination. From this experiment, we concluded that the drug caused cell death via apoptosis but not necrosis. Additionally, we observed higher apoptosis with the nano GSK treatment of the same concentration compared to their free equivalent. Although we did not observe a time dependent increase in apoptosis, we observed an increase in apoptosis in cells that received nano GSK treatment.

**Figure 11:**
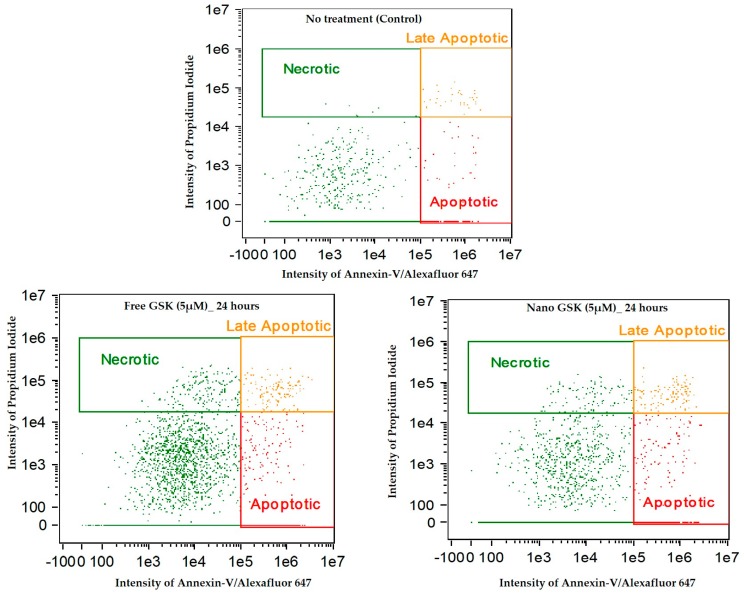

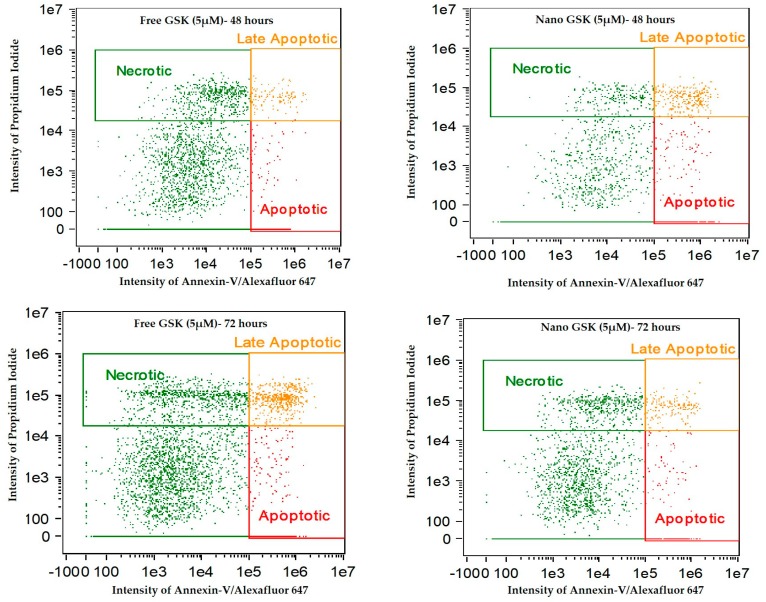
Annexin-V/Alexa Fluor™ 647 and Propidium iodide stained U87-MG cells post treatment with free and nano GSK (5 µM). Population of cells that are in early apoptosis, late apoptosis and necrosis at 24, 48 and 72 h are mentioned. The first row indicates cells that received no treatment.

Figure 11 indicates the intensity dot plots with the cell population in apoptosis, late apoptosis and necrosis. This graph pictorially represents the gradual shift of cells from one stage of cell death to another, with time.

We observed a time dependent cell killing via apoptosis. During these 72 h, the cells that were undergoing apoptosis entered late apoptosis and those undergoing late apoptosis entered secondary necrosis. This shift of population with time explains that cell death due to the free or nano GSK was mediated via apoptosis, but necrosis. Additionally, this experiment confirms the killing pattern of the 5 µM concentration of GSK (free and nano form) that was observed in cytotoxicity assay.

## 4. Discussion

Site-specific drug delivery and release are vital in cancer and nanotechnology has helped address this using targeted drug delivery and release of drug around the tumor. Nanoparticles can enhance the delivery and uptake of drugs into cancer cells thereby improving their efficacy in killing cancer cells. Reducing the efficacious dose of the drug can lower side effects thereby enhancing the therapeutic index of the drug. This study aligns well with previous studies showing that nanoparticles increase the efficacy of a specific drug and facilitates better cellular uptake compared to the unencapsulated drug, proving our hypotheses right. We achieved cytotoxicity at nanomolar concentrations of the drug only with a combination of efficient nano carrier and a targeted drug that can block the cell cycle. Reducing the effective dose of GSK by nanoparticle encapsulation, as demonstrated in this study, can help reduce the side-effects typically associated with this drug. When this research work is translated to an in vivo setting, nanoparticle characteristics that lead to higher probability of crossing the bbb (a significant barrier to drug delivery to the brain) such as a hydrodynamic diameter of around 120 nm, the surface charge around −25 mV will ensure better drug delivery to the site of action by avoiding rapid macrophage uptake. The optimal size of nanoparticles for crossing the bbb is not well established in the literature. A PDI of 0.115 indicates a fairly monodisperse nano-formulation without any aggregates, which was also visually confirmed in TEM and SEM images. We optimized the ratios of PLGA and PLAGA-PEG for our in vitro work, to consistently obtain nanoparticles with high encapsulation of GSK, smaller size and a zeta potential that imparts colloidal stability to the formulation. Nanoparticles with less negative charge can be less stable under storage conditions and may cause increased protein adsorption once they interact with serum leading to opsonization. Additionally, the prepared nano-formulation exhibited stability at 37 °C for seven days meaning that the polymer slowly disintegrates via surface erosion. Further, in vitro drug release studies have shown a gradual release of the drug over six hours in physiologically relevant media. With the two EC50 values of GSK observed in the U87-MG cell line, we established that the lower EC50 dosage when encapsulated in nanoparticles exhibited significantly higher cytotoxicity after 72 h of incubation with a *p*-value < 0.05 indicating these results are significant compared to that of free GSK. This decreases the necessity of administering higher doses of drug when efficient drug delivery systems are synthesized to encapsulate targeted drugs. Exposure to the drug over time resulted in cancer cell death causing the morphologies of the U-87 MG cells to be more spherical as opposed to the spindle shaped morphology observed in non-treated cells.

In conclusion, we have demonstrated that lower concentrations of GSK (in the nanomolar range) encapsulated in PLGA-PEG nanoparticles, enhance the cytotoxicity to U87-MG cells compared to free GSK at much higher concentrations. Despite the drug release observed within 6 h, we have the confirmed the action of GSK to be time dependent, thereby increasing the cytotoxicity of the drug over incubation time. With these experiments, we have shown that drug encapsulated nanoparticles can increase drug uptake and minimize toxicity issues and therefore reduce unwanted side effects. Additionally, we have confirmed that encapsulating the drug in the synthesized nanoparticles does not impede the activity of the drug, but rather, enhances the toxic efficacy in cancer cells. We further confirmed the mechanism of cell death triggered by free and nano GSK in U87-MG cells to be via apoptosis and not necrosis by conducting an apoptotic assay (Figure 10 and Figure 11). In this time dependent study, we showed that almost at every time interval, the percentage of cells undergoing early and late apoptosis was always higher than those undergoing necrosis. Secondary necrosis observed in the experiment was due to the cells undergoing necrosis after the onset of the late apoptotic stage [25].

Current in vitro systems lack the heterogeneity of the tumor microenvironment and thus do not have endocrine and sometimes paracrine signaling [26]. Hence, in vitro systems do not entirely represent in vivo models. While in vitro models can set expectations for in vivo results, the predictions do not prove 100% accurate. Although GSK was shown to be promising in tumor cells with a loss of function of p53, we tested and showed the efficacy of nano-encapsulated GSK in U87-MG that possesses a wildtype p53 to monitor the drug effects [27]. Future work may include testing the efficacy of GSK in the free and nano forms in p53 mutant cell line in vitro and in vivo models of GBM. As an extension of the current work, nanoparticles can be functionalized with a monoclonal antibody or a peptide ligand for tumor-specific targeting and tested in vivo to observe their treatment efficacy in mice using a relevant model of GBM.

## Figures and Tables

**Table 1:** Characterization parameters of GSK encapsulated in PLGA nanoparticles and those with different ratios of PLGA-PEG.

Nanoparticle Type	Average Size (nm)	P.D.I	Average Zeta Potential (mV)	% Encapsulation Efficiency
PLGA (100%) (n = 3)	75.49 ± 3.087	0.143 ± 0.0045	−35.56 ± 2.796	No encapsulation
PLGA-PEG (50:50) (n = 3)	157.2 ± 5.583	0.250 ± 0.0067	−27.9 ± 7.07	9.22 ± 5.32
PLGA-PEG (75:25) (n = 9)	120.5 ± 2.483	0.115 ± 0.004	−25.6 ± 0.451	56.29 ± 4.319

## Supplementary Materials

The following are available online at http://www.mdpi.com/2306-5354/5/4/83/s1, Figure S1: UV spectrum of GSK461364. (1A) Maximum wavelength (λmax) observed at 272 nm and 305 nm when dissolved in ethanol (1B) λmax observed at 311 nm when the drug is dissolved in 1% triton-x; Figure S2: EC50 curve of GSK461364 in U87-MG cell line: The graph plots % cell viability on the Y-axis when dosed with concentrations (X-axis) from 1 picomolar to 17 µM of GSK. Fit was done using dose-response simulation in Dr Fit© using the biphasic fitting type with two inflexion points.
