# Supplementary material for: GSK461364A, a Polo-Like Kinase-1 Inhibitor Encapsulated in Polymeric Nanoparticles for the Treatment of Glioblastoma Multiforme (GBM)
Source: Bioengineering (Basel). 2018 Oct 9;5(4):83. doi: 10.3390/bioengineering5040083 (PMC6315921; doi:10.3390/bioengineering5040083)
Supplement: Supplementary file 1 [file bioengineering-05-00083-s001.pdf]

Supplementary Information:

(1A) (1B)

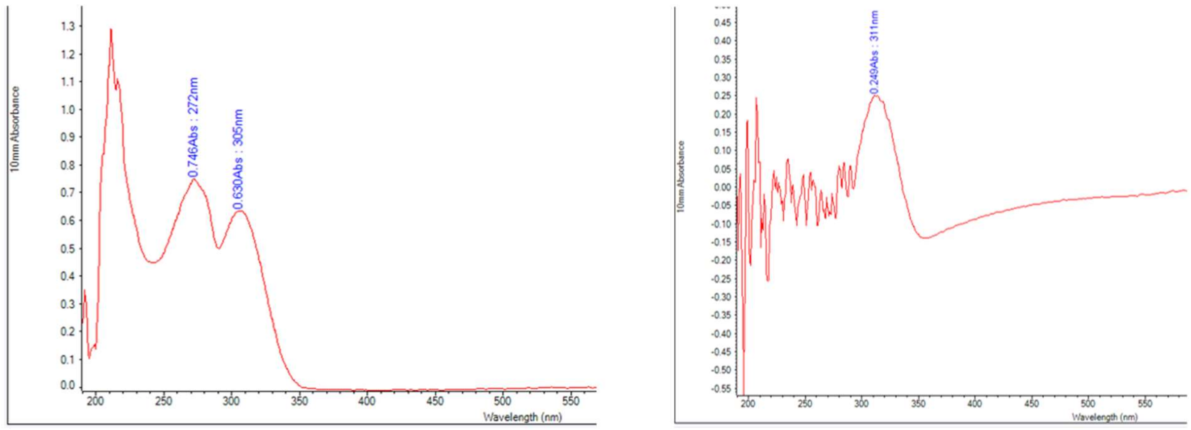

Figure S1. UV spectrum of GSK461364. (1A) Maximum wavelength (λmax) observed at 272 nm and 305 nm when dissolved in ethanol (1B) λmax observed at 311 nm when the drug is dissolved in 1% triton-x solution.

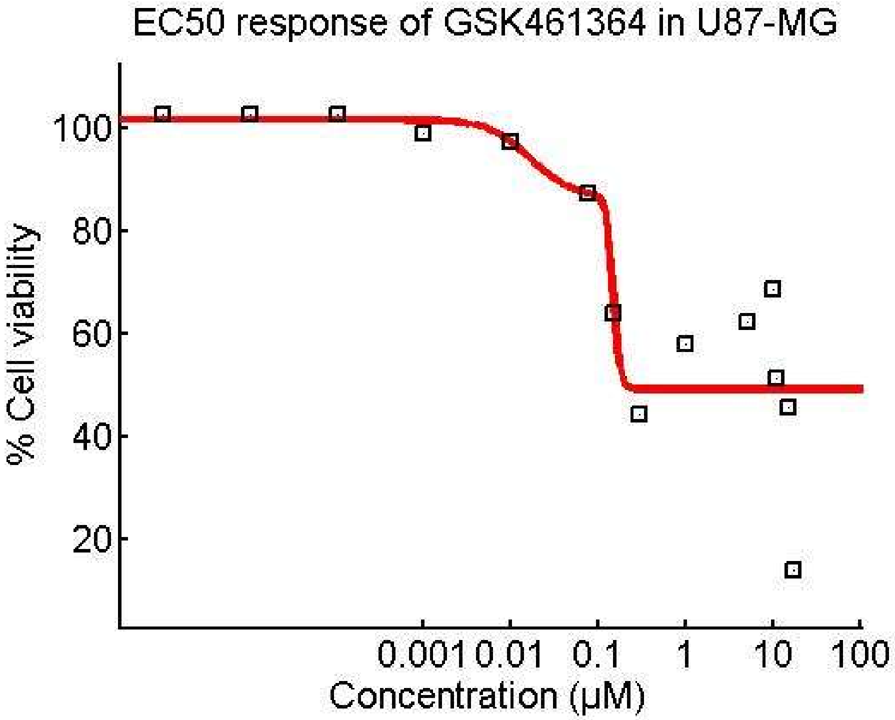

Figure S2. EC50 curve of GSK461364 in U87-MG cell line: The graph plots % cell viability on the Y-axis when dosed with concentrations (X-axis) from 1 picomolar to 17 μM of GSK. Fit was done using dose-response simulation in Dr Fit© using the biphasic fitting type with two inflexion points.
